# Chronic Physical Activity for Attention Deficit Hyperactivity Disorder and/or Autism Spectrum Disorder in Children: A Meta-Analysis of Randomized Controlled Trials

**DOI:** 10.3389/fnbeh.2020.564886

**Published:** 2020-10-22

**Authors:** Meiqi Zhang, Zhan Liu, Hongtao Ma, Daniel M. Smith

**Affiliations:** ^1^Department of Physical Education and Health Education, Springfield College, Springfield, MA, United States; ^2^School of Arts, Beijing Sport University, Beijing, China

**Keywords:** ADHD, ASD, executive function, motor skills, physical activity, children

## Abstract

**Purpose:** To explore the effects of physical activity (PA) intervention on executive function (EF) and motor skills (MS) among children with attention deficit hyperactivity disorder and/or autism spectrum disorder (ASD).

**Methods:** Relevant studies were sourced from PubMed, Web of Science, EMBASE, Cochrane Library, CNKI and Wanfang Data. Only randomized controlled trials (RCT) were included based upon the following criteria: (1) participants were children and clinically diagnosed with ADHD/ASD, (2) intervention strategies were identified as chronic physical activity, and (3) EF (e.g., cognitive flexibility) and/or MS (e.g., gross motor skills) were measured at baseline and post-intervention and compared with an eligible control group.

**Results:** Eleven studies involving 346 participants were finally identified. PA elicited significant improvements in EF and MS in children with ADHD/ASD. Regarding changes in the EF of participants, PA showed a great improvement in overall EF [standardized mean difference (SMD): 0.90, 95% confidence interval (CI) 0.49–1.30, *p* < 0.00001], inhibitory control (SMD: 1.30, 95% CI 0.58–2.02, *p* = 0.0004) and cognitive flexibility (SMD: 0.85, 95% CI 0.42–1.29, *p* = 0.0001), but no significant improvement in working memory (SMD: 0.28, 95% CI −0.15–0.71, *p* = 0.20). Significant improvements were also found with respect to gross motor skills (SMD: 0.80, 95% CI 0.30–1.30, *p* = 0.002), but no significant changes were found in fine motor skills (SMD: 0.30, 95% CI −0.91–1.52, *p* = 0.62).

**Conclusion:** Chronic PA interventions may promote EF and MS in children with ADHD/ASD, especially in inhibitory control, cognitive flexibility, and gross motor skills. However, PA interventions seemed to have insignificant effects on working memory and fine motor skills to children with ADHD/ASD.

**PROSPERO registration number:** CRD42019118622

## Introduction

Attention deficit hyperactivity disorder (ADHD) and autism spectrum disorder (ASD) are the most common neurodevelopmental disorders in children (American-Psychiatric-Association, 2013). Epidemiological studies showed that the current estimated prevalence for ADHD is ~5% of children (Polanczyk et al., 2007, 2014; Smith et al., 2016) and for ASD is 1.5–1.68% of children (Baio, 2014; Lyall et al., 2017) worldwide. Children with ASD have apparent deficits in social communication and repetitive patterns of behaviors (Fombonne, 2009), while children with ADHD have manifested symptoms such as difficulty paying attention, excessive activity, or difficulty controlling behavior in a developmentally inappropriate manner (Dunn and Kronenberger, 2003; Cormier, 2008; Lange et al., 2010; American-Psychiatric-Association, 2013). These problems are significantly detrimental to their quality of life (Ross, 2006; Mayes et al., 2008) and even persist in their later life (Klassen et al., 2004; Agranat-Meged et al., 2005; Birnbaum et al., 2005). Although there are significant differences in the core symptoms, the similarities between ADHD and ASD have been supported by clinical studies (Simonoff et al., 2008; Murray, 2010; Grzadzinski et al., 2011; Hanson et al., 2013; Craig et al., 2016; Gordon-Lipkin et al., 2018).

Impairments of cognitive and behavioral competencies (Gapin and Etnier, 2010) in ADHD and ASD are common and need to be thoroughly addressed, especially executive function (EF) performance and motor competence. EF comprises a series of self-regulatory cognitive processes, such as monitoring and controlling both thought and goal-directed behaviors (Diamond, 2013; Craig et al., 2015, 2016). Overall EF impairments have been considered as central deficits in ADHD/ASD; children within the group exhibited weakness in inhibitory control, cognitive flexibility, and working memory (Courchesne et al., 1994; Hughes et al., 1994; Ozonoff, 1995; Pascualvaca et al., 1998). Also, EF performance is closely related to motor skills (MS) because both of the disorders have similar underlying processes, which include sequencing, monitoring, and planning (Roebers and Kauer, 2009; Yazd et al., 2015). MS can be classified into gross motor and fine motor skills, which are necessary for activities in daily living. A range of MS impairments have been identified in children with ADHD/ASD (Bhat et al., 2011; Pan, 2014); this might be related to the insufficient level of neurotransmitters (Kaiser et al., 2015), which indicated the critical need for interventions to promote optimal motor and overall development.

EF and motor development have been shown to be promising endophenotypes in ASD/ADHD (Russell, 1997; Biederman et al., 2004; Willcutt et al., 2005), which depends on lifestyle and strategies. Physical activity (PA) is defined as any bodily movement produced by skeletal muscles that results in energy expenditure (Caspersen et al., 1985; DePauw and Gavron, 2005), with regular and adequate levels of PA leading to improve both cognitive function (Pontifex et al., 2013; Piepmeier et al., 2015; Benzing et al., 2018) and motor skills (Fisher et al., 2005; Wrotniak et al., 2006) for children. The Centers for Disease Control and Prevention in the U.S also recommends that children should participate in moderate to vigorous-intensity physical activity at least 60 min per day and high-intensity exercise at least three times per week (Piercy et al., 2018). However, compared with typically developing children, relatively less attention has been directed to the study of PA in children with ADHD/ASD. Given that previous studies in typically developing children generally reported PA benefits EF and MS, especially in inhibitory control (Berenguer et al., 2018) and gross motor development (Wrotniak et al., 2006; Lopes et al., 2011), PA has the potential to be used as a strategy to improve EF and MS in children with ADHD/ASD.

In summary, current literature has provided valuable information on the effects of PA on EF and MS in children with ADHD/ASD. However, it remains unclear specifically how PA affects EF and MS of children with ADHD/ASD that would be useful to clinicians and informative for future research. Therefore, the purpose of this study was to investigate the effects of PA on EF and MS in children with ADHD/ASD and explore the mechanisms of applying PA to EF and MS based on the meta-analytic findings.

## Methods

This meta-analysis is reported according to the Preferred Reporting Items for Systematic reviews and Meta-analyses (PRISMA). This study is registered with PROSPERO (CRD42019118622) and the protocol has been published in a peer-reviewed journal.

### Search Strategy

We performed a search of PubMed, Web of Science, EMBASE, Cochrane Library, CNKI and Wanfang Data from inception to August 2020. We considered all the English and Chinese studies. We used key phrases and Medical Subject Heading (MeSH) terms as follows: *physical activity, exercise, executive function, cognitive function, inhibitory control, inhibition, working memory, cognitive flexibility, motor skill, gross motor skill, fine motor skill, motor behaviors, motor functions, ADHD, ADD, and ASD*. In addition, the reference lists of included studies were examined for other potentially eligible studies.

### Inclusion and Exclusion Criteria

Articles were eligible for inclusion if the study design was a randomized controlled trial (RCT); if the participants were children and adolescents with ADHD and/or ASD; if the studies reported at least one outcome of interest measured at pre- and post-intervention. In these studies, the experimental group received a chronic PA intervention program with no limitation on the types, frequency, and intensity, while the control group was treated by sedentary resting or received no treatment. The outcomes of interest consisted of three subfunctions of EF (i.e., working memory, inhibitory control, and cognitive flexibility) and two subtypes of motor skills (i.e., gross motor skills and fine motor skills).

### Data Extraction

The titles and abstracts of studies were initially screened by two independent review authors. Related studies were recorded and managed using Endnote software. Following this, the two authors put their screened studies together and determined suitable studies with the inclusion criteria. When a disagreement happened, a third author participated to solve the problem by discussion to make a final consensus. For the studies that met the inclusion criteria, full articles were obtained for further analysis. The two authors separately extracted data from the published works using standard data extraction forms. Any inconsistencies in the process of data extraction were solved by checking original texts and reaching an agreement through discussion. Information on trial design, characteristics of the subjects, PA protocol, and relevant results were noted according to a redesigned form. We recorded the name of the first author and the year of publication for each article; the sample sizes, the ages, and gender of participants; the measurements; and the interventions for each group. When data were insufficient or inapplicable, we attempted to contact the authors by e-mail. A total of two authors were contacted (Yazd and Liu), and only Liu replied.

### Risk of Bias Assessment and GRADE Assessment

The Cochrane Collaboration's tools were used to check the random sequence generation, allocation concealment, blinding, incomplete outcome data, selective reporting and other bias. Each item was determined as a high-risk, low-risk, or unclear grade by the two authors. The grading of evidence quality and risk of bias by two authors were compared, and the third reviewer was consulted if the consensus was not attained.

### Data Analysis and Synthesis

We used Revman 5.3 (The Cochrane Collaboration, Software Update, Oxford, UK) to conduct the data analysis. The continuous outcomes were expressed as mean difference (MD) with 95% Confidence Intervals (CIs). When the MD of the outcomes is large or the unit is different, the standardized mean difference (SMD) was used. The SMD and 95% CI were calculated and then interpreted as suggested by Cohen (2013): 0.00–0.19 (trivial); 0.20–0.49 (small); 0.50–0.79 (moderate); and ≥0.80 (large).

If *p* > 0.10, I^2^ < 50%, it will be considered that heterogeneity is low enough and a meta-analysis can be conducted with a fixed-effect model. If *p* < 0.10, I^2^ > 50%, it will be considered as a high level of heterogeneity, and a random effect model will be used.

## Results

### Search Results

**Figure 1:**
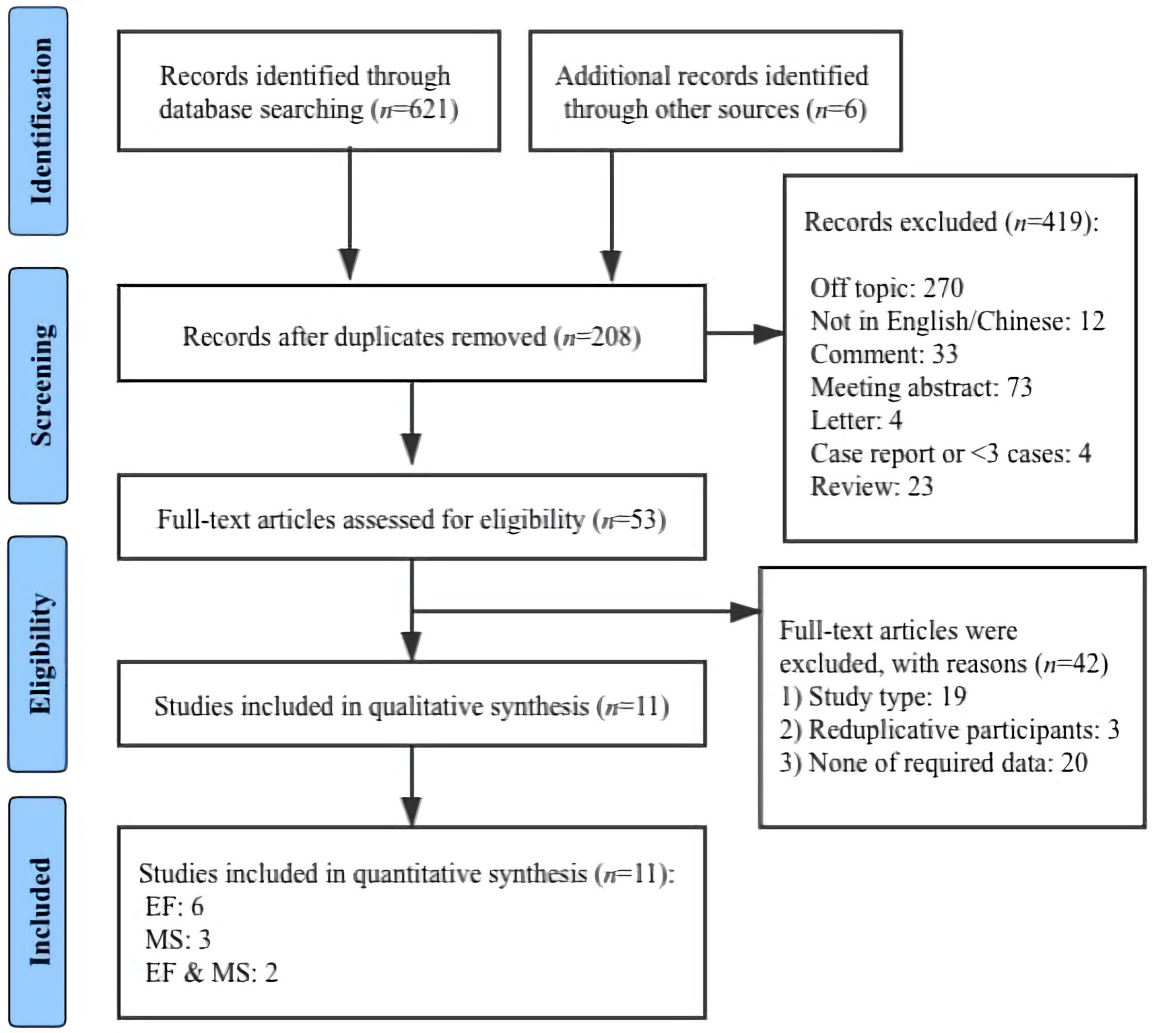
Flowchart of selecting progress.

The flow diagram illustrating the search and screening process is shown in Figure 1. The initial database search identified a total of 627 articles. After duplicates removed, 208 articles were further identified and screened, and 419 non-relevant articles were excluded. A total of 53 remaining articles were read in full text. At this point, 42 additional articles were excluded for the following reasons: (1) study types, (2) reduplicative participants, (3) natural observations, (4) animal studies, (5) none of required data. Eleven papers were rendered as a final sample.

### Characteristics of Included Trails

A total of 346 participants were included in the analysis, of which 174 (about 50.28%) participants underwent a PA intervention. The characteristics of subjects, type of intervention, intensity and duration, and measurements from the included studies were described in Table 1. The PA intervention ranged from 6 weeks to 1.5 year in duration and occurred one to five times per week. The duration of per session of PA intervention ranged from 30 to 90 min per session.

**Table 1:** Characteristics of the included trials.

**References**	**Main characteristic of the subjects**	**ADHD/ASD**	**Instruments**	**Intervention arm**	**Control arm**
Srinivasan et al. (2015)	PA: mean age was 7.88 ± 2.56, *n* = 12, 83.3% male; CON: mean age was 7.36 ± 2.02, *n* = 12, 91.7% male.	ASD	MS: BOT-2	Rhythm PA (45 min/session, 4 times/week); 8 weeks	None
Tse et al. (2019)	PA: mean age was 10.11 ± 1.20, *n* = 19, 73.7% male; CON: mean age was 9.81 ± 1.17, *n* = 21, 85.7% male	ASD	EF: False alarm error, Digit span backward	Basketball skill learning (45 min/session, 2 times/week); 12 weeks	None
Pan et al. (2017)	PA: mean age was 9.68 ± 1.61, *n* = 11, 100% male; CON: mean age was 8.49 ± 1.76, *n* = 11, 100% male	ASD	EF: WCST; MS: BOT-2	Integrated PA (70 min/session, 2 times/week);12 weeks	Waitlist
Bustamante et al. (2016)	PA: mean age was 9.4 ± 2.2, *n* = 19, 68% male; CON: mean age was 8.7 ± 2, *n* = 16, 69% male	ADHD	EF: Stop-signal inhibition task, Automated working memory assessment system	Physical games + modified aerobic sports (90 min/session, 5 times/week); 10 weeks	Sedentary control
Liu and Yang (2018)	PA: *n* = 32, 50% male; CON: *n* = 32, 50% male	ADHD	EF: Corsi block tapping test	Orienteering activity (35 min/session, 3 times/week); 14 weeks	None
Pan et al. (2019)	PA: mean age was 9.08 ± 1.43, *n* = 15, 100% male; CON: mean age was 8.9 ± 1.66, *n* = 15,100% male	ADHD	EF: WCST; Stroop test MS: TGMD-2	Table tennis + group games + conditioning training (70 min/session, 2 times/week); 12 weeks	None
Yazd et al. (2015)	PA: 6~12 years, *n* = 12, 83.3% male; CON: 6~12 years, *n* = 12, 83.3% male	ADHD	MS: BOT-2	Motor training + drug therapy (3 times/week);6 weeks	Drug therapy
Mirzaei and Aslankhani, 2015	PA: mean age was 9.5, *n* = 4, 100% male; CON: mean age was 9.5, *n* = 4, 100% male	ASD	MS: TGMD-2	Motor exercises (45 min/session, 1 time/week); 12 weeks	None
Kadri et al. (2019)	PA: mean age 14.5 ± 3.5, *n* = 20, 90% male; CON: mean age 14.2 ± 3, *n* = 20, 90%male	ADHD	EF: Stroop test	Taekwondo exercise + regular PE classes (50 min/session, 2 times/week); 1.5 year	Regular PE classes
Benzing and Schmidt (2019)	PA: mean age was 10.46 ± 1.3, *n* = 11, 86.4% male; CON:10.39 ± 1.44, *n* = 12, 81.8% male	ADHD	EF: Flanker task, Color span backwards MS: German motor test	Exergaming (30 min/session, 3 times/week); 8 weeks	Waitlist
Memarmoghaddam et al. (2016)	PA: mean age was 8.31 ± 1.29, *n* = 19, 100% male; CON: mean age was 8.29 ± 1.31, *n* = 17, 100% male	ADHD	EF: Stroop test, Go/No go test	Aerobic exercise + goal directed exercise (90 min/session, 3 times/week); 8 weeks	None

*PA, physical activity; CON, control group; RCT, randomized control trail; MS, motor skills; EF, executive function; WCST, Wisconsin card sorting test; BOT-2, Bruininks-Oseretsky Test of Motor Proficiency; TGMD-2, Test of Gross Motor Development-2*.

### Risk of Bias Among the Selected Articles

**Figure 2:**
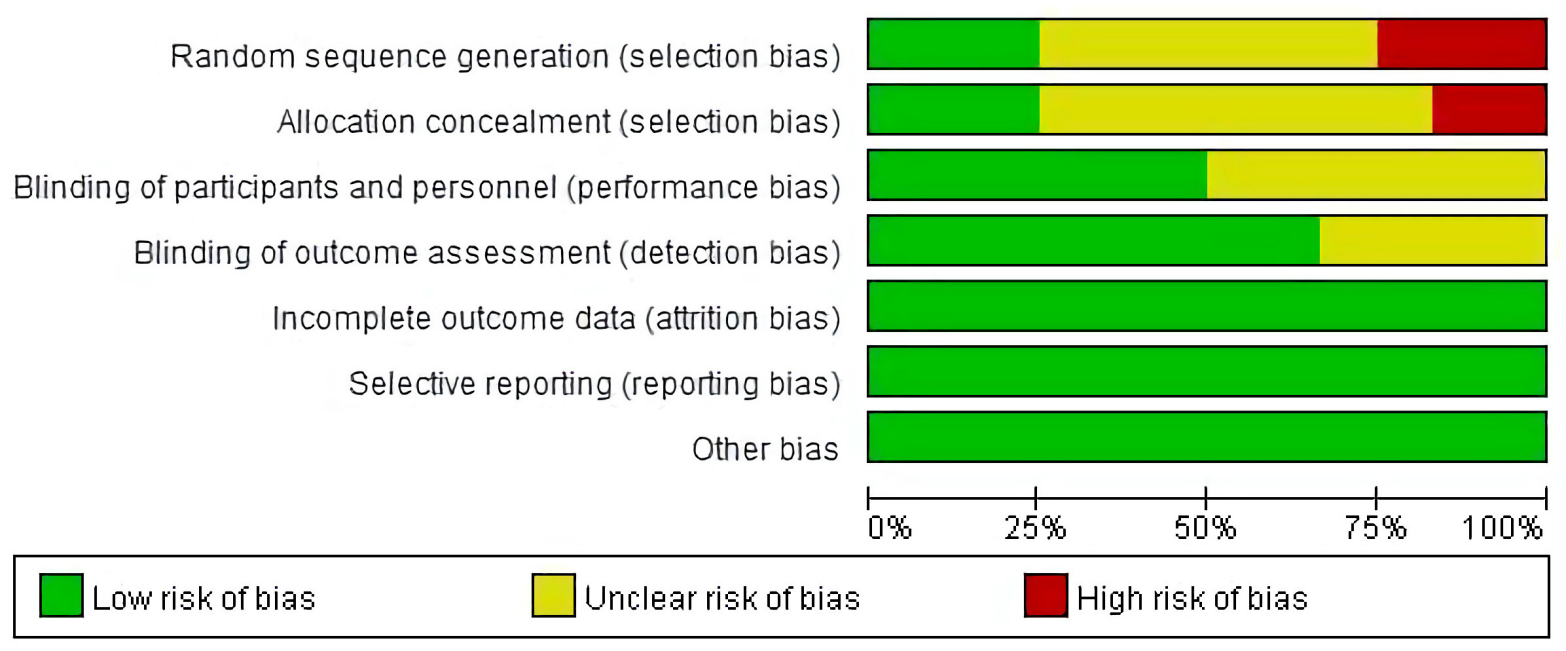
Risk of bias assessment for the included studies.

The evaluation results are shown in Figure 2.

### Executive Function

**Figure 3:**
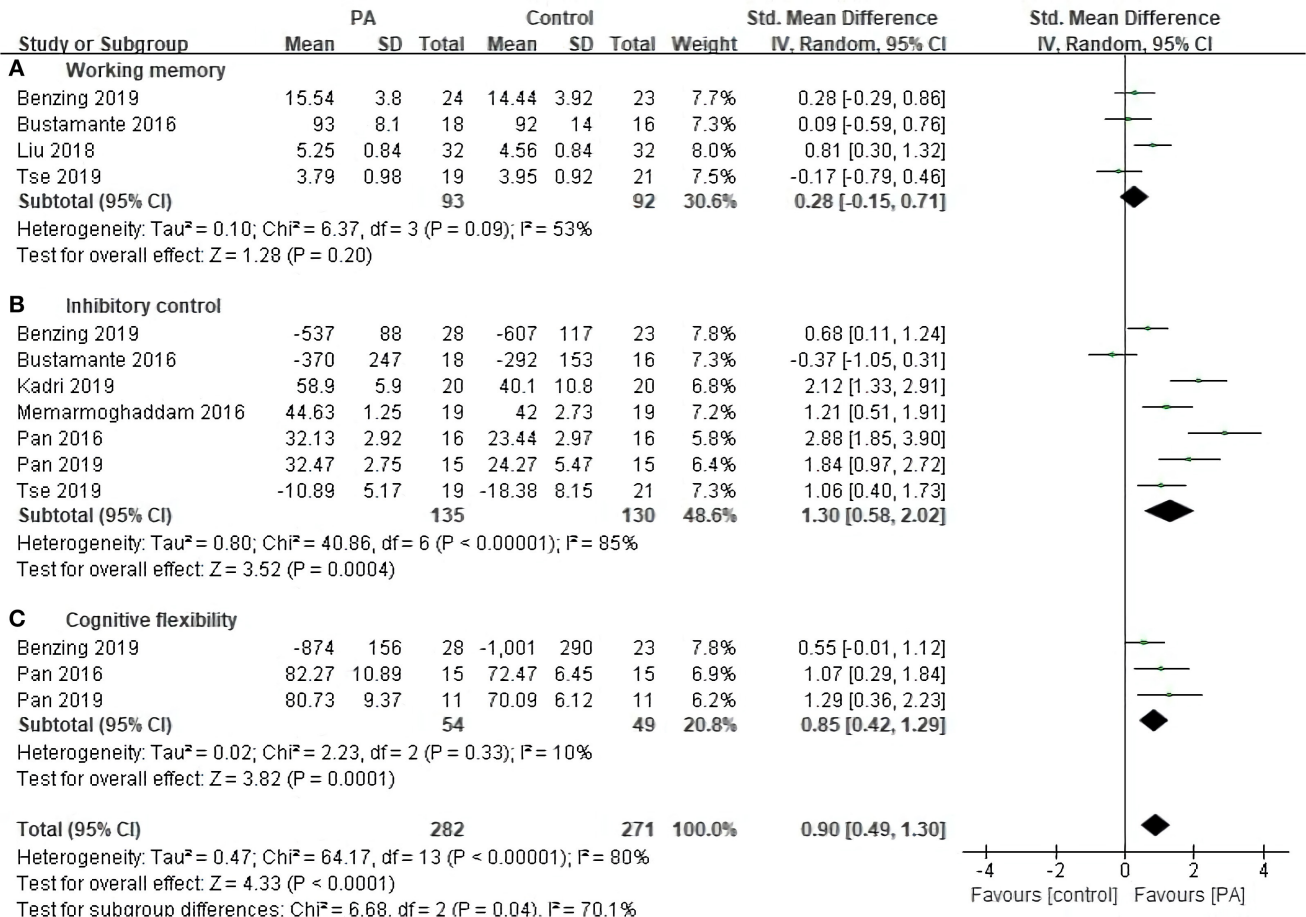
Forest plot for change in executive function. **(A)** Working memory **(B)** Inhibitory control. **(C)** Cognitive flexibility.

Eight studies with 329 subjects assessed executive function as outcomes (Figure 3). The pooled SMD of overall EF was 0.90 (95% CI 0.49–1.30, *p* < 0.0001), with high heterogeneity (*I*^2^ = 80%, *p* < 0.00001). Regarding the subfunctions of EF, the SMD was 0.28 (95% CI −0.14–0.71, *p* = 0.19) for working memory, with moderate heterogeneity (*I*^2^ = 53%, *p* = 0.09); 1.30 (95% CI 0.58–2.02, *p* = 0.0004) for inhibitory control, with large heterogeneity (*I*^2^ = 85%, *p* < 0.00001); and 0.85 (95% CI 0.42–1.29, *p* = 0.0001) for cognitive flexibility, with low heterogeneity (*I*^2^ = 9%, *p* = 0.33).

### Motor Skills

**Figure 4:**
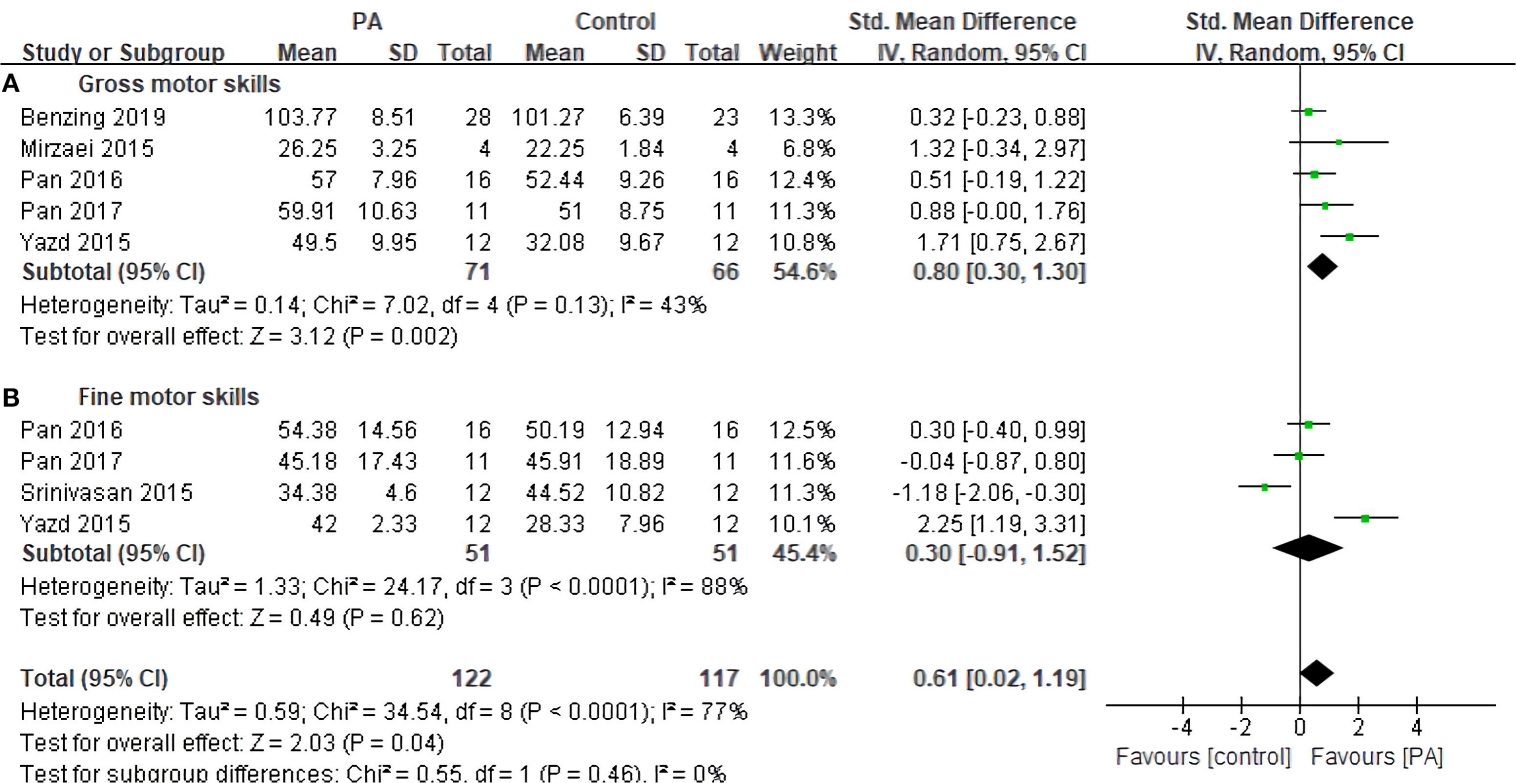
Forest plot for change in motor skills. **(A)** Gross motor skills. **(B)** Fine motor skills.

Six studies with 161 subjects assessed motor skills as outcomes (Figure 4). The pooled SMD of overall MS was 0.61 (95% CI 0.02–1.19, *p* = 0.04), with moderate heterogeneity (*I*^2^ = 77%, *p* < 0.0001). Regarding the subfunctions of MS, the pooled SMD was 0.80 (95% CI 0.30–1.30, *p* = 0.002) for gross motor skills, with small heterogeneity (*I*^2^ = 43%, *p* = 0.18); and the SMD was 0.30 (95% CI −0.91–1.52, *p* = 0.62) for fine motor skills, with large heterogeneity (*I*^2^ = 88%, *p* < 0.0001). Unfortunately, there was no significant change in fine motor skills as compared with control groups.

## Discussion

The present meta-analysis revealed that PA could significantly improve EF and MS of ADHD/ASD children in terms of cognitive flexibility, inhibitory control, and gross motor skills. However, our analysis did not provide strong evidence to the effectiveness of PA on working memory and fine motor skills in ADHD/ASD children.

Our findings are aligned with previous studies on the efficacy of PA on improving some aspects of cognition of individuals with ASD/ADHD (Cerrillo-Urbina et al., 2015; Tan et al., 2016) and are consistent with the results of motor skills from the systematic reviews (Den Heijer et al., 2017). The clinical PA programs, however, are shown to be relatively less efficacious in the magnitude of improvements in the working memory compared with earlier results of a systematic review (Suarez-Manzano et al., 2018) and a previous RCT study (Smith et al., 2016). Also, we did not find a significant improvement in fine motor skills, which is not consistent with the results from another recent systematic review (Jeyanthi et al., 2019).

Deficits in EF are believed to be an important origin of ADHD/ASD symptoms (Diamond, 2013). Available evidence, however, indicates that some deficits in executive function could be improved by PA intervention as some RCTs (Smith et al., 2016; Jeyanthi et al., 2019) and meta-analysis (Xue et al., 2019) show. Our work extends the results of previous studies and finds that PA interventions are beneficial to inhibitory control and cognitive flexibility in children with ADHD/ASD. The underlying mechanisms of PA-induced EF improvements might be related to two aspects, which are the promotion of attention allocation (especially in the dorsolateral prefrontal cortex; DLPFC) and changes in the concentration of neurotransmitters. Children who participate in PA are more likely to be affected by contextual interference, which the interference in performance and learning that arises from performing one task in the context of other tasks. For example, in the exercise of table tennis (Benzing et al., 2018), the children had to continuously modify their body direction and location to effectively catch the ball in one scenario but need to lob the ball in another. These non-predetermined and rarely repeated tasks place higher demands on executive processes (Carey et al., 2005). Thus, the processing of pertinent information is likely to lead to greater learning. In addition, EF is associated with the prefrontal cortex, mainly on the frontal pole, ventrolateral prefrontal cortex (VLPFC), and DLPFC (Diamond, 2013). Children with ADHD/ASD were identified with significantly unstable neurotransmitters system and lower concentration of monoamine neurotransmitters like dopamine (Dresel et al., 2000; Krause et al., 2004) and norepinephrine (Bymaster et al., 2002), which may lead to hypo-arousal level (Jeyanthi et al., 2019) in PFC. Strong evidence reveals that PA is effective in stimulating arousal levels in prefrontal cortex and activating neurotransmitter systems like dopamine (Foley and Fleshner, 2008), which may explain our results that PA intervention could promote EF in children with ADHD/ASD.

Notably, contrary to previous studies (Hillman et al., 2005; Kamijo et al., 2011; Koutsandréou et al., 2016), this study showed that PA intervention had favorable effects on overall EF in children with ADHD/ASD; the effects of PA on working memory, however, were still limited. Similar to our findings, a recent meta-analysis of RCTs by Xue et al. (2019) identified the minor benefits of PA interventions on working memory. The inconsistent results could partly be explained by the differences among intervention methods, as well as the difference in the baseline of participants. Further researchers would need to consider more about the intervention program design (e.g., types of PA, intensity, frequency, and duration) and the characteristics of children (especially with respect to age and the severity of disorders), which may impact trial results.

Delayed motor development has been widely found in children with ADHD/ASD (Thapar and Cooper, 2016). To children with ADHD/ASD, impaired MS are not only impacting their daily activities, but it is also a barrier to social interaction and community integration with peers (Pan et al., 2017). Our findings are strongly supported by most of the reviewed studies (Pan, 2014; Pan et al., 2017) that overall MS improved after PA interventions. Several brain regions are believed to be crucial for acquiring and executing skilled motor behaviors including cerebellum, basal ganglia, and regional motor cortex of the frontal lobe (Ungerleider et al., 2002; Luft and Buitrago, 2005; Halsband and Lange, 2006), and neurotransmitter system. In addition, the theory about arousal level as aforementioned might also be used to explain the mechanism. As the previous study demonstrated that there were negative correlations between physical activity and brain arousal level on motor competence, especially with lower performance, in children with ADHD (Berger, 2012).

A potential reason for the no significant changes in fine motor skills in children with ADHD/ASD after physical activity interventions might be related to variability in the intensity of PA interventions. Given that psychostimulant drugs (e.g., Methylphenidate) have demonstrated positive effects on fine motor skills in children with ADHD/ASD by modulating neurotransmitters, fine motor skills could be improved with increasing neurotransmitter levels. Moderate to high-intensity PA has also been recognized as an effective way to enhance the level of neurotransmitters (Vučković et al., 2010; Lin and Kuo, 2013) and the plasticity of the central nervous system (CNS). However, all the included studies in this meta-analysis mentioned the duration and frequency of interventions but did not report the exercise intensity used in the studies, which may partially account for the null findings regarding fine motor skills. Additionally, molecular studies (Ferguson and Cada, 2004; Yu et al., 2010) reported gross motor performance appears to be normal in ADHD but there were structural deficits displayed in fine motor skills, which might further explain our findings. Previous fMRI evidence showed that complex finger movements could activate more domains in the brain (e.g., SM1, SMA, PMA, PFC, SPC, cerebellum, and basal ganglia) than simple finger movements (Chang et al., 2002). However, most of the interventions included in our study were fundamental movements (e.g., treadmill training, exergames). Although we cannot simply conclude by the findings in animal models, it is still reminding that future studies and clinical applications should consider adding more exercise about fine motor skills in training programs.

To the best of our knowledge, this meta-analysis is the first study to quantitively compare the effectiveness of chronic PA and non-intervention on EF (e.g., working memory, cognitive flexibility, and inhibitory control) and MS (e.g., gross motor skills and fine motor skills) in children with ADHD/ASD. One strength of the study is the strict inclusion principles, which could increase the validity of causal inferences. Also, subgroup analysis was conducted to explore the detailed changes of physical activity on motor skills and executive functions in children with ADHD/ASD might be another important feature of the study.

However, some limitations were still present in the evaluation. First, the different measurements of the included studies may lead to high heterogeneity in this meta-analysis. Second, a relatively small number of studies in motor skills in comparison to EF studies included in the review may affect the findings of our analysis. As a result, the effects of PA on MS remain to be determined and further explored. Third, the results of the meta-analysis might be limited by the lack of large sample, multi-center and long-term studies in the included studies, which may have some limitations in guiding clinical applications.

In conclusion, the present meta-analysis supports the positive effects of PA on EF and MS in children with ADHD/ASD, especially in inhibitory control, cognitive flexibility, and gross motor skills. These findings indicate that PA can be implemented in children with ADHD/ASD as an alternative training modality. However, we found insignificant effects of PA interventions on working memory and fine motor skills in children with ADHD/ASD. Future studies should investigate the effects of PA in ADHD/ASD longitudinally through multicenter RCT with large sample sizes.

## Data Availability Statement

All datasets generated for this study are included in the article/supplementary material.

## Author Contributions

This study was conceptualized by MZ, ZL, HM, and DS. DS contributed to collecting data. Analyzing data and drafting the manuscript was by MZ and ZL. HM contributed to revising and approving the final version of the manuscript. All authors contributed to the article and approved the submitted version.

## Conflict of Interest

The authors declare that the research was conducted in the absence of any commercial or financial relationships that could be construed as a potential conflict of interest.

## Abbreviations

ADHD: Attention Deficit Hyperactivity Disorder
ASD: Autism Spectrum Disorder
BOT-2: Bruininks-Oseretsky Test of Motor Proficiency, Ed.2
CI: Confidence Intervals
CNS: Central Nervous System
SM1: Contralateral Primary Somatosensory Cortex
DLPFC: Dorsolateral Prefrontal Cortex
EF: Executive Function
GRADE: Grading of Recommendations Assessment, Development
MD: Mean Difference
MS: Motor Skills
MABC-2: Movement Assessment Battery for Children
PA: Physical Activity
PMA: Pre-Motor Area
RCT: Randomized Control Trials
SMD: Standardized Mean Difference
SMA: Supplementary Motor Area
TGMD-2: Test of Gross Motor Development-2
VLPFC: Ventrolateral Prefrontal Cortex
PM1: Primary Sensorimotor Cortex
PFC: Prefrontal Cortex
SPC: Superior parietal lobule.
